# Validation of a novel patient reported tool to assess the impact of treatment in erythropoietic protoporphyria: the EPP-QoL

**DOI:** 10.1186/s41687-021-00345-7

**Published:** 2021-08-03

**Authors:** G. Biolcati, S. Hanneken, E. I. Minder, N. J. Neumann, J. H. P. Wilson, P. J. Wolgen, D. J. Wright, A. J. Lloyd

**Affiliations:** 1 Centre for Porphyrias, Istituto Dermatologico S. Gallicano - Istituti Fisioterapici Ospitalieri, Rome, Italy; 2 Private Practice Empoderm, Düsseldorf, Germany; 3 Stadtspital Triemli, Porphyria Outpatient Clinics, Zurich, Switzerland; 4 Department of Dermatology, Heinrich Heine University, Duesseldorf, Germany; 5 The Department of Internal Medicine, Center of Lysosomal and Metabolic Diseases, Erasmus Medical Center, Rotterdam, Netherlands; 6 Clinuvel Pharmaceuticals Limited, Melbourne, Australia; 7 Acaster Lloyd Consulting Ltd, London, UK

**Keywords:** Erythropoietic protoporphyria, EPP-QoL, Patient reported outcomes, Psychometric validation

## Abstract

**Background:**

A novel treatment has been developed for erythropoietic protoporphyria (EPP) (a rare condition that leaves patients highly sensitive to light). To fully understand the burden of EPP and the benefit of treatment, a novel patient reported outcome (PRO) measure was developed called the EPP-QoL. This report describes work to support the validation of this measure.

**Methods:**

Secondary analysis of trial data was undertaken. These analyses explored the underlying factor structure of the measure. This supported the deletion of some items. Further work then explored the reliability of these factors, their construct validity and estimates of meaningful change.

**Results:**

The factor analyses indicated that the items could be summarised in terms of two factors. One of these was labelled EPP Symptoms and the other EPP Wellbeing, based on the items included in the domain. EPP Symptoms had evidence to support its reliability and validity. EPP Wellbeing had poor psychometric properties.

**Conclusions:**

Based on the analysis it was recommended to drop the EPP Wellbeing domain (and associated items). EPP Symptoms, despite limitations in the development of items, showed evidence of validity. This work is consistent with the recommendations of a task force that provided recommendations regarding the development, modification and use of PROs in rare diseases.

## Introduction

Erythropoietic protoporphyria (EPP) is a rare metabolic disease characterized by abnormally elevated levels of protoporphyrin IX in erythrocytes (red blood cells) and plasma [1, 12]. When exposed to light in the visible spectrum, protoporphyrin IX is activated resulting in severe phototoxicity [12]. EPP patients show sensitivity to visible rather than UV light and it manifests in painful phototoxicity. Symptoms tend to occur within a few minutes of skin exposure to light/sunlight, and can take hours or days to resolve. Repeated episodes of phototoxicity can result in altered skin appearance with permanent changes (e.g. skin thickening with a waxy or leathery appearance) [8]. EPP can greatly impact on patients’ health related quality of life (HRQL), including daily and social activities and personal relationships [5, 7]. Some patients are effectively restricted to spending much of their life indoors and out of the light/sun.

CLINUVEL has developed a photoprotective agent for use in EPP that has been tested in international trials. Because of the nature of the disease and the protective effect of the treatment, a major outcome to be assessed is the impact on health related quality of life and other patient reported outcomes such as daily activities. CLINUVEL has previously used the SF-36 questionnaire [13] in earlier trials to assess the generic impact of treatment on patients’ HRQL. Other studies used the Dermatology Life Quality Index (DLQI) [4]. Neither of these measures was considered specific enough to capture the full impact of EPP on HRQL. Therefore, CLINUVEL worked with clinical experts to develop an assessment of HRQL for use specifically in patients with EPP. The resulting measure is called the EPP-QoL. The EPP-QoL was developed based on clinical expert opinion and was used in clinical trials without formal evidence of its psychometric properties. However, the trial data provide a useful resource for exploring the psychometric properties of the tool retrospectively. These analyses were planned, described in a statistical analysis plan and conducted independently from the team who conducted the trial analysis.

The first task in this analysis was to explore the underlying factor structure of the instrument. This was done to explore which items grouped together, to help identify a scoring system for the measure and to identify poorly functioning items. Further analyses then assessed the reliability and validity of the emerging domains.

## Methods

### Development of the EPP-QoL

The EPP-Qol was developed through a process of expert clinical consultation. Before the development of a modern treatment for EPP there was no standardised form of patient reported assessment. The clinical study team considered that the Dermatology Life Quality Index lacked face validity in the assessment of EPP because certain key features of the condition are not reflected in the questions. In order to develop the EPP-QoL the clinical experts held a series of round table meetings to agree on the concepts to measure and to discuss the development of the item wording. The instrument then went through rounds of review before its content was considered finalised. Informal assessments of the instrument by several groups of EPP patients were undertaken to provide feedback on the items and wording. The measure had not been formally validated prior to the initiation of this trial programme.

### Datasets

The psychometric validation was conducted on data collected from two trials: trial CUV029 (conducted over a 9 month period in Europe and included 6 site visits) and trial CUV030 (conducted over a 6 month period in the US and included 4 site visits).

### Measures

#### Erythropoietic protoporphyria-quality of life (EPP-QoL)

The EPP-QoL was designed to assess quality of life in EPP patients. It was originally designed as a unidimensional instrument that included 15 items measuring various aspects of quality of life, including the impact of EPP on well-being, ability to partake in social/leisure/outdoor activities on sunny days, the choice of clothes worn on sunny days, and issues around transportation. The items are scored using a 4-point Likert scale ranging from 0 to 3, − 3-0, or − 2-1 depending on the item. The scale is scored additively, resulting in a maximum of 35 and a minimum of − 10. The higher the score, the more the quality of life is impaired. The recall period for the EPP-QoL is 2 months. The instrument was provided to patients for completion every 2 months during the clinical trials. Completion rate was 93%.

### Analyses

The approach to the psychometric analysis was informed by Fayers & Machin [3] and Revicki et al. [9]. The focus of the psychometric analysis was on exploring the psychometric structure of the instrument through factor analysis. Following this, the performance of the identified domains was examined in terms of reliability and validity. Reliability assessments explored measurement error. The validity assessments were designed to determine what the domains were measuring and how changes in the domain scores can be interpreted. The analyses were limited in their scope because this was secondary analysis of clinical trial data which meant that the analyses were constrained to available data collection points and variables.

#### Exploratory factor analysis

An exploratory factor analysis (EFA) was used to identify the structure of the EPP-QoL (e.g. whether the unidimensional structure could be supported). Factors with an eigenvalue of 1 or more were extracted. Different rotations were examined, including oblique and orthogonal approaches. The suitability of the data for conducting an EFA was assessed on the Kaisser-Mayer-Olkin Measure of Sampling Adequacy and Bartlett’s Test of Sphericity.

#### Item performance

The performance of each item of the EPP-QoL was assessed to explore the frequency of responses (to identify heavily skewed items or items with large floor or ceiling effects). A skewed distribution of responses was defined where fewer than 10% of responses occurred in two adjacent scale points. This was used to highlight problematic items [10].

#### Instrument review

Each item was reviewed in terms of poor functioning based on the findings from the EFA and the item analysis. Evidence of poor functioning items was based on whether the item did not fit the emerging factor structure; or there was evidence of skew, floor or ceiling effects or high rates of missing responses. If there was substantial evidence that an item was functioning poorly then the item could be removed from the measure. The factor analysis suggested that the items grouped into two broad domains that were labelled EPP Symptoms and EPP Wellbeing (see below). Analyses of reliability and validity therefore explored the performance of these two domain scores.

#### Reliability

Internal consistency (Cronbach’s alpha) was estimated for EPP Symptoms & EPP Wellbeing using data from visit 3 only. To explore test-retest reliability of the scale, data were analysed from the middle period of the trial (visits 3 and 4). Participants were considered to be stable if they had experienced no phototoxicity prior to visit 3 and visit 4. The intra-class correlation coefficient (ICC) and Pearson’s correlation were estimated for the EPP Symptoms and EPP Wellbeing domain scores.

#### Construct validity

The performance of the EPP Symptoms and Wellbeing domains was tested in terms of its relationship to other markers of disease severity or outcomes as well as other measures of HRQL. This analysis focused on the Dermatology Life Quality Index (DLQI) which is the most widely used patient reported measure of health status or HRQL used in dermatology. However, it is a generic dermatology tool not specific to EPP and has not been specifically validated in EPP patients to the best of our knowledge.

The DLQI data can be used to express an overall impact of the dermatological condition on the patients’ life quality. Cut-off scores have been published (0–1 No effect on patients’ life; 2–5 small effect; 6–10 moderate effect; 11–20 very large effect; 21–30 extremely large effect) [6]. Participants were divided into groups based upon these cut-off scores and the differences in EPP scores were estimated. The EPP-QoL domain scores were also benchmarked against the severity of recent phototoxicity episodes as rated by the patient in a diary (Table 1).

**Table 1:** Description of the severity of phototoxicity reactions as recorded in the trials

	Phototoxicity severity
**0 None**	No symptoms.
**1–3 Mild**	The reaction is transient and easily tolerated, not requiring any treatment.
**4–6 Moderate**	The reaction caused discomfort and interrupted usual activities. Some form of treatment was required.
**7–9 Severe**	The reaction caused considerable interference with usual activities and may have been incapacitating, requiring treatment.
**10 Worst Imaginable**	The reaction causes extensive interference with usual activities and is incapacitating, requiring treatment and/or hospitalisation.

#### Sensitivity

The sensitivity of the EPP scores was estimated in terms of effect sizes. The EPP scores were explored to test the extent to which they changed as a result of a phototoxicity event. Simple effect sizes were estimated for patients who reported moving from experiencing some photoxicity at Visit 3 to experiencing none at Visit 4.

#### Minimal important difference

The trial data included two subjective markers of health status that were used as anchors to estimate minimal change – the DLQI and peak phototoxicity severity. These variables were used as a proxy for the degree of difference between groups that would be considered important. The MID estimates were calculated as the arithmetic difference between mean values for different groups of patients defined either in terms of peak phototoxicity or DLQI grades.

## Results

### Factor structure

A varimax exploratory factor analysis was conducted to identify the structure of the EPP-QoL. Kaisser-Mayer-Olkin Measure of Sampling Adequacy was 0.95 and Bartlett’s Test of Sphericity was significant (*p* ≤ 0.001), suggesting the data were suitable for EFA. Two factors were consistently identified in different iterations of the analysis and explained a total of 69.4% of the variance (Table 2). The factor analysis identified that ten items loaded on Factor 1, three items on Factor 2; and two items did not load on either dimension. Item 2 showed a weak loading on Factor 1, and items 3 and 9 showed weak loadings on Factor 2. A Promax EFA conducted to confirm the findings of the varimax analysis, reported very similar results.

**Table 2:** Mean (and standard deviation, 95% confidence intervals) of EPP-QoL domain scores separated by degree of impact of EPP (determined by DLQI score)

	DLQI Impact	N	Mean	Std. Deviation	95% Confidence Interval for Mean	
					Lower	Upper
**EPP Symptoms**	Moderate effect	23	46.6	22.1	37.0	56.2
	Very large effect	45	73.1	21.9	66.5	79.7
	Extremely large effect	8	82.9	20.3	65.9	99.8
**EPP Wellbeing**	Moderate effect	23	17.4	7.9	14.0	20.8
	Very large effect	45	13.3	7.6	11.0	15.6
	Extremely large effect	8	14.6	5.9	9.7	19.5

N.b. Data from the only patient classified as *Small effect* on DLQI is not shown

Reviewing the items that load on each domain suggests that Factor 1 items could be described in terms of EPP severity and the impact of disease (and so this domain was labelled EPP Symptoms). Factor 2 includes items relating to the broader impact on quality of life and well-being (and was termed EPP Wellbeing). Items 2 and 9 did not load on either factor. Items 2, 10 and 13 showed a skewed pattern of responses. Seven items showed evidence of floor or ceiling effects (2, 8, 10, 11, 12, 14 & 15). Item 3 cross-loaded on both Factors 1 & 2, and was not conceptually coherent with the other items in the Factor 2 domain. Based on this analysis, items 2, 3 and 9 were dropped from the instrument. With these items removed a repeated EFA explained 77% of the variance.

### Data distribution

**Fig. 1:**
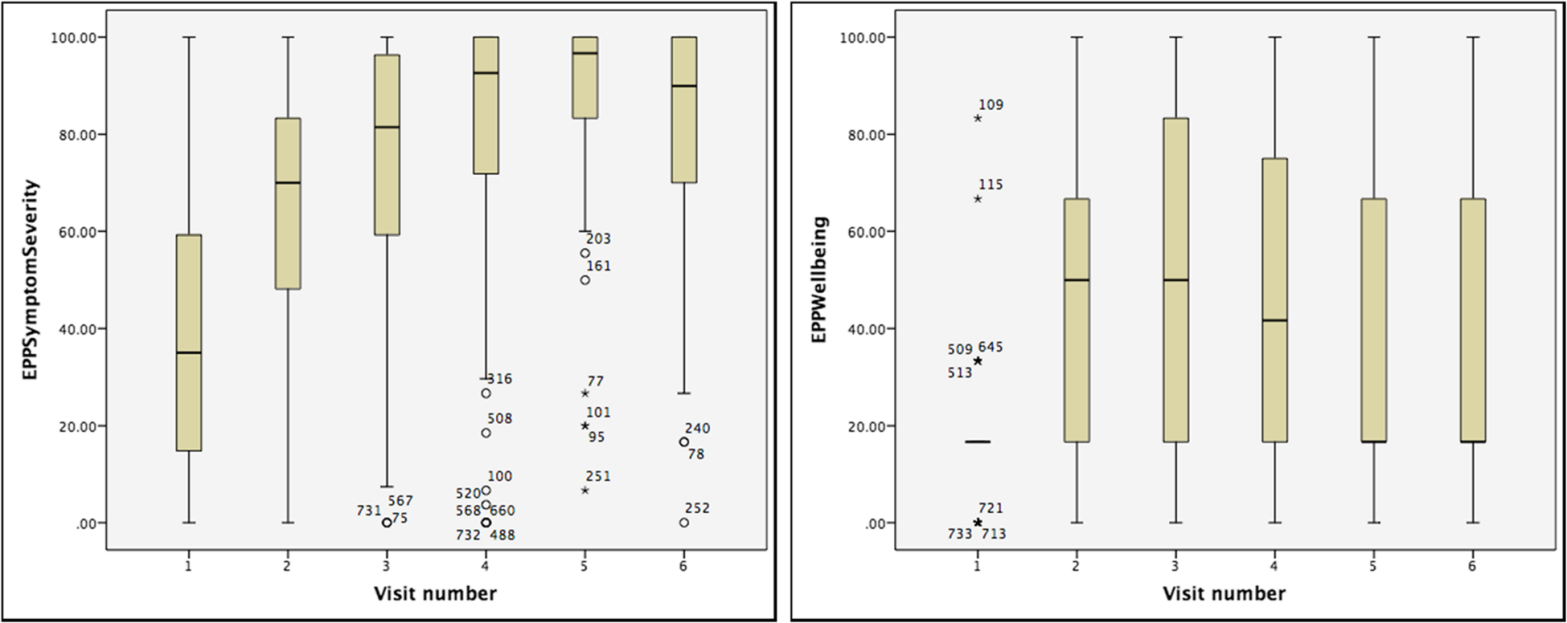
**a** and **b** EPP Symptoms and EPP Wellbeing over time. Plots show median (broad bar), interquartile range or IQR (brown box), 1.5 times IQR (whiskers), circles and stars are outliers

Figure 1a & b show the distribution of EPP Symptoms and Wellbeing domain scores over time from the trial data. These analyses are collapsed across trial arms. The Symptoms domain shows an improvement in scores over the course of the trial. The Wellbeing domain shows no evidence of a change in scores over time, and a clustering of data below 20 (at baseline).

### Scale reliability

Both subscales show high (and acceptable) internal consistency [11]. Table 3 also shows estimates of the test-retest reliability of the two domain scores over time in the total sample and a defined stable patient group. Table 4 shows the influence of each individual item on internal consistency. The EPP-Symptoms domain shows some evidence of stability; the ICC is at the lower end of what would be considered acceptable. The EPP-Wellbeing domain shows poor test-retest reliability when only stable patients are included.

**Table 3:** Internal consistency and test-retest reliability for the EPP-Qol subscales

		Total sample visit 3–4		Stable only patients		
	Cronbach’s alpha	ICC	Pearson’s r	ICC	Pearson’s r	T test
EPP Symptoms	0.954	0.809	0.680	0.543	0.373	t = 1.91, n.s.
EPP Wellbeing	0.880	0.831	0.711	0.192	0.115	t = −1.56, n.s.

**Table 4:** Item-total correlations and Cronbach’s alpha with item deletion for the EPP Symptoms score (Cronbach’s alpha for scale =0.954) from Visit 3 only

	Corrected Item-Total Correlation	Cronbach’s Alpha if Item Deleted
Item 4: Over the last 2 months, how much has EPP influenced the choice of the clothes you wear on a sunny day?	0.762	0.951
Item 5: Over the last 2 months, how often did you feel you were at risk of developing EPP symptoms?	0.707	0.953
Item 6: Over the last 2 months, how much has EPP affected any social or leisure activities on a sunny day?	0.854	0.947
Item 7: Over the last 2 months, how much has EPP influenced your need to plan before leaving your house?	0.803	0.949
Item 8: Over the last 2 months, has EPP limited your ability to undertake activities in a spontaneous manner?	0.875	0.946
Item 10: Over the last 2 months, how much has EPP interfered with your going shopping or looking after your home (indoors and outdoors) or garden on a sunny day?	0.813	0.949
Item 11: Over the last 2 months, how much has EPP preventing you from attending outdoor social activities with family and friends?	0.871	0.947
Item 12: Over the last 2 months, how much has EPP limited your amount of outdoor activities?	0.885	0.946
Item 13: Over the last 2 months, how often did you experience typical EPP skin complaints?	0.720	0.952
Item 15: Over the last 2 months, how much has EPP influenced your method of transportation or seating preference during transportation?	0.754	0.951

### Construct validity

#### Benchmarking EPP-QoL against DLQI

The construct validity of the EPP-QoL domain scores was tested by benchmarking against the DLQI. The DLQI data showed that all patients (bar one) were classed as having at least a moderate effect of EPP on their lives. There is no difference in the EPP Wellbeing domain scores for patients in the different severity level groups. In contrast, there is a clear linear change in the EPP Symptoms score. The mean EPP Symptom score is 46.6 for moderate effect, 73.1 for very large effect and 82.9 for extremely large effect (Table 2), (F = 9.23; *P* < 0.0001). DLQI total score is significantly correlated with EPP Symptom domain (*r* = 0.52; *P* < 0.0001), but not the EPP Wellbeing domain (*r* = − 0.10; n.s.).

#### Benchmarking EPP-QoL against the experience of recent phototoxicity?

The trial participants were grouped according to peak level of phototoxicity in the previous 60 days. There is no significant difference between groups defined by peak photosensitivity severity for the EPP Wellbeing score (Table 5). In contrast the EPP Symptom score shows a linear change in scores with increasing severity of groups defined in terms of the phototoxicity variable (F = 51.5, *P* < 0.0001).

**Table 5:** EPP-QoL domain scores with respect to peak toxicity severity from previous 60 days

	Peak toxicity severity	N	Mean	***Std. Deviation***	***95% Confidence Interval for Mean***	
					***Lower Bound***	***Upper Bound***
EPP Symptoms	None	150	5.5	*10.9*	*3.8*	*7.3*
	Mild	109	24.6	*19.8*	*20.9*	*28.4*
	Moderate	67	39.5	*25.7*	*33.2*	*45.8*
	Severe	26	42.6	*27.4*	*31.5*	*53.6*
EPP Wellbeing	None	150	43.6	*30.9*	*38.6*	*48.5*
	Mild	109	44.6	*33.1*	*38.4*	*50.9*
	Moderate	67	43.3	*31.7*	*35.6*	*51.0*
	Severe	26	41.0	*28.4*	*29.6*	*52.5*

N.b. Data from only patient classified as *Worst imaginable* in terms of peak sensitivity is not shown

#### Minimal important difference (MID)

MID estimates were calculated for differences in EPP-Symptoms score based on the differences between sub-groups defined by the DLQI and the 60 day peak phototoxicity severity (Table 6). An average MID value was estimated, which was a change of 13 points. It was not possible to estimate an MID for differences on the EPP-Wellbeing because none of the differences between sub-groups were statistically significant.

**Table 6:** Estimates of important difference on EPP-Symptoms scale based on benchmark criteria from DLQI and 60 day peak phototoxicity severity

EPP-Symptoms	Benchmark difference	Difference estimate
**DLQI criteria**	Moderate – Very large effect	26.5
	Very large – Extremely large effect	9.8
**60 day peak phototoxicity severity**	None – mild	5.5
	Mild –Moderate	19.1
	Moderate – Severe	14.9
	Severe – Worst imaginable	3.1

## Discussion

Many people advocate the use of condition specific outcome measures to really understand the nature of the impact of a disease [2]. In a condition like EPP there is evidence from patient testimonies that patients simply stay inside to avoid light exposure and resulting phototoxicity. This may have a very significant effect on patients’ psychological state and social wellbeing. Phototoxicity may be avoided, but clearly people are unable to live an otherwise normal life. Therefore, measuring the impact of EPP using a dermatology specific instrument may capture the impact of skin lesions but will likely miss the wider social and psychological impact of EPP and so will underestimate the disease burden. This led the team to try to develop a novel instrument to assess the burden of EPP.

Rare diseases present some significant challenges for researchers concerned with the development and use of patient reported outcomes (PROs). In a prevalent disease, a new PRO can be developed using in depth qualitative research with people affected by the disease followed by large scale psychometric research to understand the measurement properties of the new instrument. In rare diseases, this approach to development is much harder because of the difficulty in recruiting sufficient numbers of patients. In the present study the team relied upon expert opinion to guide the content of the instrument. Anecdotally the EPP-QoL was well received by patients, but no formal cognitive interviewing was conducted to support this, which is a limitation. The initial trial work has been used to explore the measurement properties of the instrument. Based upon these analyses the team has made substantial changes to the instrument items and scoring.

The present report describes psychometric analysis of this instrument using available trial data. The team recognises the limitations in the development of the measure and so where the psychometrics suggest that changes to the instrument could improve it, then these have been actioned. The work that has been undertaken suggested that the measure reflected two underlying domains – EPP Symptoms and EPP Wellbeing. However, based on a review of all of the analyses the team has concluded that only the EPP Symptoms domain should be taken forward as a measure. The EPP Wellbeing domain has been dropped. In broad summary, the EPP Symptom score has good internal consistency, and test-retest reliability. The EPP Symptom score reflected differences in DLQI scores and peak photosensitivity reactions supporting its construct validity. An MID was estimated as a 13 point change. More work is needed to explore MID and also to establish was might be considered a meaningful change threshold. In future clinical studies the use of a smaller MID estimate (5–6 points) could be tested if only very mildly affected patients are included. This should be stated a priori and it should be tested.

Rare disease trials also present problems with the use of PRO measures. PRO data are subjective, prone to biases and so the resulting data can have high error variance. Large studies can detect the therapeutic signal against the background noise using statistical analysis. In rare diseases, clinical trials are usually small, and so the interpretation of the PRO data and change from baseline in scores is therefore more complex. A guidance paper from the International Society for Pharmacoeconomics and Outcomes Research (ISPOR) recommends that in rare disease trials it is preferable to use disease specific measures [2]. In addition, they state that evidence of the reliability, construct validity and the ability to detect change should be evaluated and documented. They also recognise how challenging it is to develop new PROs in rare diseases. Where there is interest in developing a measure in a rare disease, they outline the simplified methods that could be used, reflecting the limited availability of patients. The present study is perhaps an example of this pragmatic approach recommended by ISPOR. The development of the tool had some limitations, but the availability of data from the trials allowed the team to heavily edit the instrument content based on evidence.

Other study limitations should also be noted. The psychometric analysis was based on secondary use of trial data. This meant that the analyses could only use available data and available time points. For example test retest reliability was assessed over a 2 month time window (in stable patients) which was probably too long a time period for the assessment of test retest reliability. This is partly due to the fact that the measure has a 2 month recall period and afamelanotide is dosed every 2 months. The 2 month recall period may be a limitation of the measure. This allows patients to reflect over a period of time, which is useful considering that phototoxic events are infrequent (although milder reactions can be more common and can disrupt activities of daily living). The long recall period introduces a greater risk of measurement bias. Also, the trial data did not allow us to examine the validity of the measure in different cultural contexts. More work is needed to establish criterion validity and an estimate of what would be considered a meaningful change threshold. The exploratory factor analysis that identified the two underlying factors in the EPP-QoL was only based on Eigen values. Other methods also exist, such as the Hull method, for identifying underlying factors. Quite a high proportion of items showed evidence of floor or ceiling effects. Several of these items had a high frequency of “not at all” responses – often to questions about the limitations on aspects of life that people with EPP experienced. This may be evidence of poorly designed items. It should also be clear that the EPP-Qol was developed to support the trial programmes for afamelanotide, a treatment developed by the study sponsor. It was developed by clinical experts in EPP to capture the impact of the condition. This psychometric analysis was restricted to data from two clinical trials. The EPP-QoL has been used in other clinical trials, notably a US trial CUV039 the data from which could also be explored in future analyses. Lastly it is possible that the psychometric properties of this instrument vary with seasonality. This again could be explored in future research. Because of different study limitations we believe that this report should not be considered the definitive psychometric analysis of the EPP-QoL, but rather an analysis that explores aspects of the performance of the measure based on available data from the trials.

In conclusion we report a case study of the development of a new PRO in a very rare condition – EPP. The measure was tested and adapted in order to produce the best possible instrument from the original content. Lessons were learnt in this process regarding the measurement of patient benefit in a rare disease. Despite its limitations we hope that this instrument will be informative regarding the burden of EPP.

## Data Availability

Study data are not available for public use.

## Abbreviations

DLQI: Dermatology Life Quality Index
EFA: Exploratory Factor Analysis
EPP: Erythropoietic protoporphyria
EPP-QoL: Erythropoietic Protoporphyria-Quality of Life
HRQL: Health-related quality of life
ICC: Intra-class correlation coefficient
ISPOR: International Society for Pharmacoeconomics and Outcomes Research
MID: Minimal Important Difference
