# Correction to: Validation of a novel patient reported tool to assess the impact of treatment in erythropoietic protoporphyria: the EPP-QoL

**DOI:** 10.1186/s41687-021-00348-4

**Published:** 2021-08-15

**Authors:** G. Biolcati, S. Hanneken, E. I. Minder, N. J. Neumann, J. H. P. Wilson, P. J. Wolgen, D. J. Wright, A. J. Lloyd

**Affiliations:** 1 Centre for Porphyrias, Istituto Dermatologico S. Gallicano - Istituti Fisioterapici Ospitalieri, Rome, Italy; 2 Private Practice Empoderm, Düsseldorf, Germany; 3 Stadtspital Triemli, Porphyria Outpatient Clinics, Zurich, Switzerland; 4 Department of Dermatology, Heinrich Heine University, Duesseldorf, Germany; 5 The Department of Internal Medicine, Center of Lysosomal and Metabolic Diseases, Erasmus Medical Center, Rotterdam, Netherlands; 6 Clinuvel Pharmaceuticals Limited, Melbourne, Australia; 7 Acaster Lloyd Consulting Ltd, London, UK

## Correction to: Journal of Patient-Reported Outcomes (2021) 5:65 https://doi.org/10.1186/s41687-021-00345-7

Following publication of the original article [1], the authors identified an error in the author name of G. Biolcati.

The incorrect author name is: G. Biolcatti

The correct author name is: G. Biolcati

The author group has been updated above and the original article [1] has been corrected.
